# Variations in T Cell Transcription Factor Sequence and Expression Associated with Resistance to the Sheep Nematode *Teladorsagia circumcincta*

**DOI:** 10.1371/journal.pone.0149644

**Published:** 2016-02-18

**Authors:** Hazel Wilkie, Anton Gossner, Stephen Bishop, John Hopkins

**Affiliations:** The Roslin Institute & R(D)SVS, University of Edinburgh, Easter Bush, Midlothian, EH25 9RG, United Kingdom; North Carolina State University, UNITED STATES

## Abstract

This study used selected lambs that varied in their resistance to the gastrointestinal parasite *Teladorsagia circumcincta*. Infection over 12 weeks identified susceptible (high adult worm count, AWC; high fecal egg count, FEC; low body weight, BW; low IgA) and resistant sheep (no/low AWC and FEC, high BW and high IgA). Resistance is mediated largely by a Th2 response and IgA and IgE antibodies, and is a heritable characteristic. The polarization of T cells and the development of appropriate immune responses is controlled by the master regulators, T-bet (*TBX21*), GATA-3 (*GATA3*), RORγt (*RORC2*) and RORα (*RORA*); and several inflammatory diseases of humans and mice are associated with allelic or transcript variants of these transcription factors. This study tested the hypothesis that resistance of sheep to *T*. *circumcincta* is associated with variations in the structure, sequence or expression levels of individual master regulator transcripts. We have identified and sequenced one variant of sheep *TBX21*, two variants of *GATA3* and *RORC2* and five variants of *RORA* from lymph node mRNA. Relative RT-qPCR analysis showed that *TBX21*, *GATA3* and *RORC2* were not significantly differentially-expressed between the nine most resistant (AWC, 0; FEC, 0) and the nine most susceptible sheep (AWC, mean 6078; FEC, mean 350). Absolute RT-qPCR on all 45 animals identified *RORVv5* as being significantly differentially-expressed (p = 0.038) between resistant, intermediate and susceptible groups; *RORCv2* was not differentially-expressed (p = 0.77). Spearman’s rank analysis showed that *RORAv5* transcript copy number was significantly negatively correlated with parameters of susceptibility, AWC and FEC; and was positively correlated with BW. *RORCv2* was not correlated with AWC, FEC or BW but was significantly negatively correlated with IgA antibody levels. This study identifies the full length *RORA* variant (*RORAv5*) as important in controlling the protective immune response to *T*. *circumcincta* infection in sheep.

## Introduction

The abomasal strongylid *Teladorsagia circumcincta* is a major cause of sheep parasitic gastroenteritis [1, 2]. The most susceptible animals are weaned lambs [3], but many eventually suppress larval development and egg production [4] through the development of IgA and IgE anti-parasite antibodies [5–7]. The ability to control infection is a heritable characteristic and most flocks consist of animals with a range of susceptibilities. Indeed, IgA levels and fecal egg counts (FEC) have been used as selectable markers for resistance [4, 8, 9] and antigens that promote the production of abomasal IgA antibodies have been identified as potential vaccine candidates [10].

The candidate gene approach for the identification of molecular markers for selection aims to evaluate the relationship between phenotype and a variation in a gene [11]. Several studies have analysed abomasal mucosa to identify genes associated with resistance to *T*. *circumcincta* or the related parasite *Haemonchus contortus* [12–14]. However, the induction of the immune response to these parasites occurs in the draining abomasal (gastric) lymph node (ALN) and the events within that node are likely to determine the quality and quantity of the response that occurs within the mucosa and the consequent clinical outcome.

This current study exploited parasite-naïve Blackface lambs with diversity in their predicted genetic resistance to *T*. *circumcincta* [15]. Both immunological [16] and microarray analyses [17] of ALN linked Th2 responses to high IgA levels, low FEC and resistance, and also showed that Th1/Th17 T cell activation in susceptible sheep resulted in granulomatous inflammation and low antibody levels that failed to control infection (high AWC and FEC). In mouse and human gastrointestinal nematode infections, resistance is determined by Th2 activation [18] associated with a balanced Th1/Th2/Treg response [19]. Uncontrolled Th1 and/or Th17 activation leads to clinical disease [20]. Consequently, the clinical outcome of infection is mediated by differential T cell activation.

The multiple effector functions of CD4 T cells are achieved by the differentiation of multipotential precursors into distinct polarized subsets, which is largely regulated by the master regulators T-bet, GATA-3 and RORγt; transcription factors that transactivate the genes and mediate the subset-specific functions [21]. T-bet (*TBX21*) promotes Th1 differentiation by transactivating *IFNG* and increasing IFNγ production, and by repressing Th2 activation [22, 23]. GATA-3 (*GATA3*) regulates Th2 development [24] by direct binding to enhancers and/or promoters of the Th2 cytokine gene cassette (*IL4*, *IL5* and *IL13*) and inducing their expression; it also inhibits Th1 development [25]. Th17 cells express neither T-bet nor GATA-3 [26]; antigen-activated naïve T cells in the presence of TGFβ and IL-6 differentiate into IL-17A producing T cells regulated by RORγt (*RORC2*) [27]. Maximum production of IL-17A also requires the related transcription factor RORα (*RORA*) [28]. RORα is also critical for the development and function of the nuocyte [29] subset of innate lymphoid cells 2 (ILC2), which are crucial in the induction of Th2 cells and the development of anti-helminth immunity [30] in mice. Tregs have also been shown to play a role in anti-helminth immunity in mice [19], however previous work in *T*. *circumcincta*-infected sheep [16] has shown that there is no differential expression of the Treg transcription factor *FOXP3* in resistant and susceptible sheep.

In addition to controlling T cell differentiation, all four transcription factors contribute to the pathogenesis of chronic inflammatory diseases. T-bet plays a role in the abnormal expression of Th1 cytokines in human Crohn’s disease [31] and GATA-3 is prominent in the development of ulcerative colitis [32]. Furthermore, *GATA3* gene variants and deletion mutants have been linked to a number of other inflammatory pathologies, including asthma and IgE-mediated allergy [33, 34]. The major function of Th17 cells is in the development of inflammatory reactions, and many inflammatory diseases have been ascribed to increased Th17 activity [35]. Consequently, RORγt and RORα have also been linked to abnormal inflammation [36, 37].

In this study we characterise the different transcript variants of the four master regulators expressed in sheep and then compare the expression of these individual variants in animals of defined resistance status. Finally we quantify expression levels of variants to enable their correlation with quantitative phenotypes of resistance to *T*. *circumcincta*.

## Materials and Methods

### Animals and experimental design

Female Blackface lambs (10–13 weeks old), from a flock previously used for quantitative genetic and QTL analyses [38], were housed in worm-free conditions. Ten lambs were sham-infected controls and 45 lambs were infected with ~2300 infective L3 *T*. *circumcincta* larvae three times a week for 12 weeks. At post mortem, two days after the last infection, the abomasal AWC ranged from 0 to 11300 and FEC from 0–950 eggs per g (S1 Table). The lambs selected for analysis were chosen to maximize the power of detecting differential expression. Consequently, animals were ranked (1–45) according to their infection level [15]. Full details of the animals, animal husbandry, infection protocols, quantitative phenotypes and population genetic analyses have been described previously [15, 16]. Animal experiments were approved by University of Edinburgh Ethical Review Committee and conducted under an Animals (Scientific Procedures) Act 1986 Project Licence.

Animals were housed in a large open barn in three pens; infected animals were in two adjacent pens of approximately 180 m^2^ each and the uninfected lambs were in a pen of approximately 50 m^2^. All animals were bedded on clean straw and had *ad libitum* access to hay and water supplemented twice daily with Maize Lamb Pellets (16.0% protein; Carrs Billington, Carlisle, UK). Animals were examined at least daily. All lambs were vaccinated with Heptavac P Plus at 5 and 6 weeks and Scabivax (both MSD Animal Health, UK) at 6 weeks. Most lambs were administered 2 ml Hexasol (Norbrook Pharmaceuticals, UK) to treat respiratory infections. Infected animals showing mild symptoms of visceral pain associated with parasite infection were treated, under veterinary instructions, with 2 ml Finadyne (MSD Animal Health). Lambs were killed by intravenous administration of Euthetal (Merial Animal Health, UK).

### Sample collection and total RNA isolation

Abomasal (gastric) lymph nodes (ALN) were removed immediately post mortem and stored at –80˚C in RNAlater (Ambion, UK). Total RNA was isolated using the Ribopure Kit (Ambion) according to the manufacturers’ instructions. Contaminating DNA was removed by On-column PureLink^®^ DNase I treatment (Ambion). The quantity, quality and integrity of the RNA samples were determined using a NanoDrop ND-1000 spectrophotometer and Agilent 2200 TapeStation system; all had an RNA Integrity Number of > 7.5.

### Cloning and sequencing of transcription factors

cDNA was synthesised from 1 μg RNA using SuperScript™ II RT with RNaseOUT (Invitrogen, UK) and oligo-dT(15) primer (Promega, UK). The predicted sequences of the full length sheep genes were obtained by NCBI-BLAST of the bovine sequences against the Oar v3.1 sheep genome assembly (http://www.livestockgenomics.csiro.au/sheep/oar3.1.php/). Primers were selected using Primer-BLAST (http://www.ncbi.nlm.nih.gov/tools/primer-blast/) and reanalysed using Net Primer (http://www.premierbiosoft.com/netprimer/). The primers used to amplify overlapping sections of genes are shown in S2 Table. Each primer set was used in RT-PCR using FastStart Taq (Roche, UK) as per manufacturers’ instructions. PCR products were fractionated by agarose gel electrophoresis, visualized by gel red /UV transillumination, purified using MinElute PCR Purification Kit (Qiagen), ligated into pGEM-T Easy vector (Promega) and transformed into JM109 High Efficiency Competent Cells (Promega). Colonies that contained the inserted vector and target sequence were incubated overnight at 37°C in LB broth with 50 μg/ml ampicillin. Purified plasmid was extracted from the culture using QIAprep Spin MiniPrep Kit (Qiagen) following the manufacturer’s protocol. Three clones from six resistant and six susceptible lambs for each insert were sequenced using T7 and SP6 primers, with BigDye® Terminator v3.1 Cycle Sequencing Kit (Applied Biosystems, UK). The GeneRacer^TM^ Kit for full-length, RNA ligase-mediated rapid amplification of 5’ and 3’ cDNA ends (Invitrogen) was used to sequence the 5’ and 3’ untranslated region (UTR) of the transcription factors. Primers and nested primers (S2 Table) were designed and the protocol was followed as detailed by the manufacturer, using SuperScript^TM^ III protocol for reverse transcription reaction and Platinum^(R)^ Taq DNA Polymerase High Fidelity protocol for the amplification of the final product, which were cloned as described above.

### RT-qPCR quantification of transcript variants

Primers for quantitative real-time RT-PCR (RT-qPCR) were designed and optimized for all gene variants (S2 Table); primer specificity was confirmed by sequencing. The *RORC2* full length RT-qPCR primers did not distinguish between *RORC2* and *RORC1*, however *RORC1* is not expressed in lymph node but is expressed in liver (S1 Fig). RT-qPCR was performed in 15 μl volumes containing 7.5 μl FastStart Universal SYBR Green Master (Rox) 2x concentration (Roche), 2 μl template cDNA, 0.25–1.0 μl primers (S2 Table) and nuclease-free water. Reactions were prepared using a CAS-1200™ robot and performed on a Rotor-Gene 6 (Qiagen). Amplification (conditions in S2C Table) was followed by dissociation curve analysis. Relative expression levels were quantified in cDNA in triplicate from two separate RT-qPCR reactions for each of the nine most resistant (ranked 1–9) and the nine most susceptible sheep (ranked 37–45), and duplicate no-template controls were included in all runs. Optimized RT-qPCR assays had an efficiency between 95–105% and R^2^ value of 0.98–0.99.

Relative gene expression levels were calculated in GenEx 5 Standard Programme (MultiD Analyses AB, Sweden) using the comparative 2-(ΔΔ Cq) method and normalized to the geometric mean of *GAPDH* and *SDHA*. Fold changes were calculated from ΔCq values using GenEx. To calculate copy number of *RORAv2* and *v5* in all 45 infected animals, a standard curve of linearized plasmid DNA was used with a dynamic range that spanned at least five orders of magnitude. For each point on the standard curve, copy numbers were calculated from Cq values using the following formula:

$$\text{molecules per ng}=\left(\left[1\text{x}\;10^{\text{-}9}\right]/\left(\text{M g}/\text{mol}\right)\right]\;\text{x}\;\left[6.03\text{x}10^{\text{-}23}\;\text{molecules}/\text{mol}\right])$$

M = size of plasmid x 660g/mol per bp. The expression levels were normalized by dividing the copy number derived from the standard curve by the calculated normalization factor for each individual sample. The normalization factor was calculated using the method described by Vandesompele [39], using the geometric mean of *GAPDH* and *SDHA*. RT replicates were averaged per animal and multiplied by the dilution factor (x100) to calculate the copy number per μg of total RNA.

### Statistical analysis

Relative gene expression levels were analyzed in GenEx using an unpaired, 2-tailed t-test to determine the difference between groups. Graph Pad Prism 6 for Windows (Graph Pad Software, USA) was used for statistical analysis of the absolute expression data. The data were grouped into resistant, intermediate and susceptible (n = 15 per group) and a one-way ANOVA was performed to determine overall significance and Tukey’s multiple comparisons test (within ANOVA) was used to determine significance between groups. The correlations between transcript levels and quantitative phenotypes were analyzed with Spearman’s correlation coefficient (r_s_). P-values ≤ 0.05 were considered statistically significant.

## Results

### Identification of transcription variants of sheep transcription factors

Cloning and sequencing identified two transcription variants of *GATA3* and *RORC2* and five variants of *RORA*. *GATA3* is encoded on the plus strand of chromosome 13 (Oar v3.1; NC_019464). The difference between full length *GATA3* (LN848231) and *GATA3v1* (LN848232) is the deletion of a single codon (g.806_808delGAA) at the 5’ end of exon 3 (Chr13: 12,082,935–12,082,937) which represents the deletion of a glutamic acid (E) at position 260 within the protein (S2 Fig). *RORC2* is encoded on the minus strand of chromosome 1 (NC_019468) and, in comparison to the full length gene (LN848233), *RORC2v1* (LN848234) has a 36 bp deletion (g.1237_1272del) at the 3’ end of exon 7 (Chr1: 100,653,158–100,653,123), which represents the deletion of 12 amino acids (GKYGGVELFRAL) at position 359–370 (S3 Fig) of RORγt. Analysis of ovine *TBX21* identified only one sequence, identical to XM_004012818.2.

**Fig 1:**
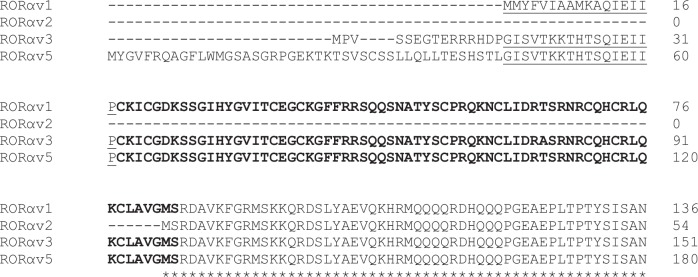
*Ovis aries RORA* transcript variants. Derived NH_2_-protein sequences of RORαv1, v2, v3 and v5. Bold is the zinc-finger DNA binding domain; underlined are B domains. The derived protein sequences of RORαv2 and RORαv4 are identical. Sequences are identical after amino acid 1 (M) of RORαv2.

*RORA* is encoded on the plus strand of chromosome 7 (NC_019464) where five transcript variants (LN848235 –LN848239) were identified in the region spanning Chr7: 46,097,028–46,731,648. All five variants are identical from position 46 of *RORAv1* (Chr7: 46,691,081), which represents the 5’ end of exon 6 (S4 Fig). The five variants have variable usage of exons 1 to 6 and consequently 5’ UTRs of different lengths (S5 Fig). *RORAv1*, *v2*, *v3* and *v5* have unique translation start sites, while *RORAv2* and *v4* have the same translation start site and encode identical derived protein sequences of 387 amino acids. *RORAv5* encodes the largest protein of 513 amino acids (Fig 1). The derived protein sequences of all five variants are identical 3’ from amino acid 83 of RORαv1.

### RT-qPCR quantification of transcription factor expression

Relative RT-qPCR assays were developed for *TBX21*, for the two variants of *GATA3* and *RORC2* and for five variants of *RORA*. These were used to compare the nine most resistant (R) and the nine most susceptible (S) lambs (S1 Table). The resistant group (rank 1–9) had no detectable AWC or FEC, mean body weight (BW) of 37 kg and high IgA antibody levels (mean 0.76 relative units). The susceptible lambs (rank 37–45) were those with the highest AWC (mean 6078, maximum 11300), high FEC (mean 350, maximum 950), low BW (mean 23.7 kg) and low IgA (mean 0.22 units). Table 1 shows the relative expression (fold change) of each transcription factor and variant between the resistant and susceptible groups and shows that all but *RORCv5* were equally expressed in the ALN of both groups. *RORAv5* was 1.57 fold higher in the resistant animals but p = 0.08. *RORCv3* could not be quantified because expression levels were too low for accurate measurement.

**Table 1:** Relative quantification of transcription factor transcripts in ALN.

Gene	Fold change R vs. S	p value
*TBX21*	-1.10	0.61
*GATA3*	1.06	0.62
*GATA3v1*	-1.12	0.47
*RORC2*	1.18	0.42
*RORC2v1*	1.12	0.54
*RORAv1*	-1.13	0.79
*RORAv2*	-1.07	0.83
RORAv4*	-1.04	0.88
*RORAv5*	1.57	0.08

* *RORAv3* signal was too low for accurate measurement.

### Correlation of transcription factor levels and quantitative phenotypes

Absolute quantitative analysis was performed on *RORAv5* as it was the only transcription factor variant that showed evidence of differential expression between the R and S groups; *RORAv2* was also quantified as the control *RORA* variant. *RORAv2* expression in all 45 animals was 162938 ± 14799 (transcript copy number per μg RNA ± SD), in comparison to 108806 ± 11211 (p = 0.0045) for *RORCv5*.

**Fig 2:**
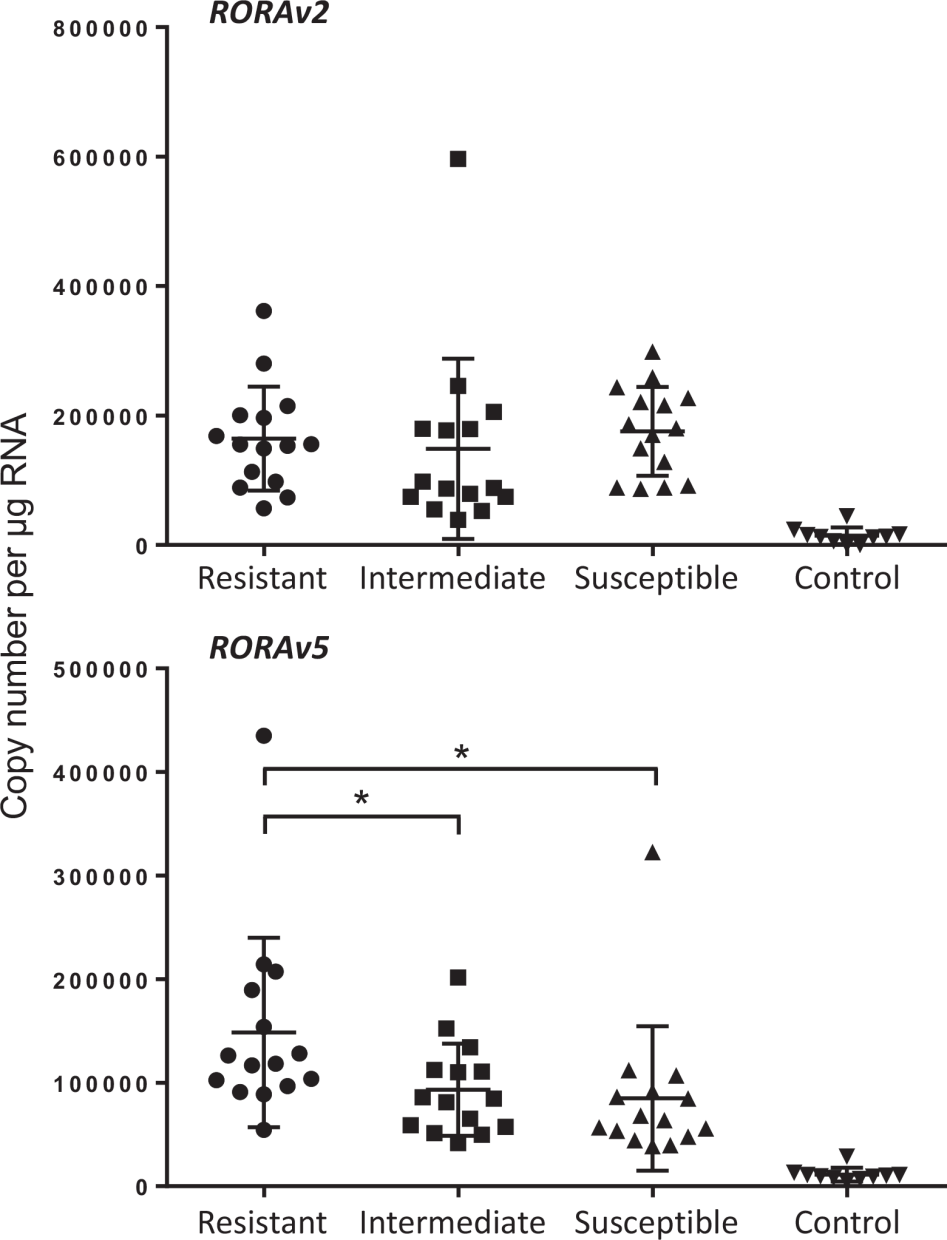
Expression of *RORAv2* and *RORAv5* in the ALN of *T*. *circumcincta* infected sheep. Copy number per μg total RNA in rank 1–15 resistant sheep (AWC 0–300, mean 59; FEC 0–25, mean 1.7); rank 16–30 intermediate (AWC 200–3100, mean 1508; FEC 0–475, mean 87) and rank 31–45 susceptible (AWC 2900–11300, mean 5167; FEC 75–950, mean 288) sheep. Error bars are means ± SD. One-way ANOVA for *RORAv2* p = 0.77, infected animals only (p <0.0003 with controls); *RORAv5* p = 0.038, infected animals only (p <0.0001 with controls); * p ≤ 0.05 (Tukey’s multiple comparison test within ANOVA). P ≤ 0.05 for all three infected groups vs. uninfected controls for both *RORAv2* and *RORAv5*.

Comparison of *RORAv2* and *RORAv5* expression in 15 most resistant (rank 1–15, mean AWC 59 and FEC 1.7), 15 intermediate (rank 16–30, AWC 1508 and FEC 87) and the 15 most susceptible (rank 31–45, AWC 5167 and FEC 288) sheep (Fig 2) shows that *RORAv2* is expressed equally in all three infected groups (164411 ± 80305, 148781 ± 139319 and 175622 ± 68746), with one-way ANOVA p = 0.77. In contrast *RORAv5* expression levels were significantly different between the infected groups (p = 0.038); mean levels in the resistant sheep were 148439 ± 91397 in comparison with the intermediate (93161 ± 44477, p ≤ 0.05) and the susceptible group (84818 ± 69870, p ≤ 0.05).

Expression levels of *RORAv2* and *RORAv5* in the uninfected controls were 14336 ± 12927 and 11308 ± 6571 respectively and not significantly different (p = 0.52). *RORAv2* was significantly up-regulated 9–11 fold in all three infected groups (p < 0.0003) in comparison with the uninfected controls, with no significant discrimination between resistant, intermediate and susceptible animals. In contrast *RORAv5* was significantly increased 13 fold (p < 0.0001) in the resistant sheep but only 7 (p ≤ 0.005) and 6 (p ≤ 0.005) fold in the intermediate and susceptible sheep, in comparison to the controls.

**Fig 3:**
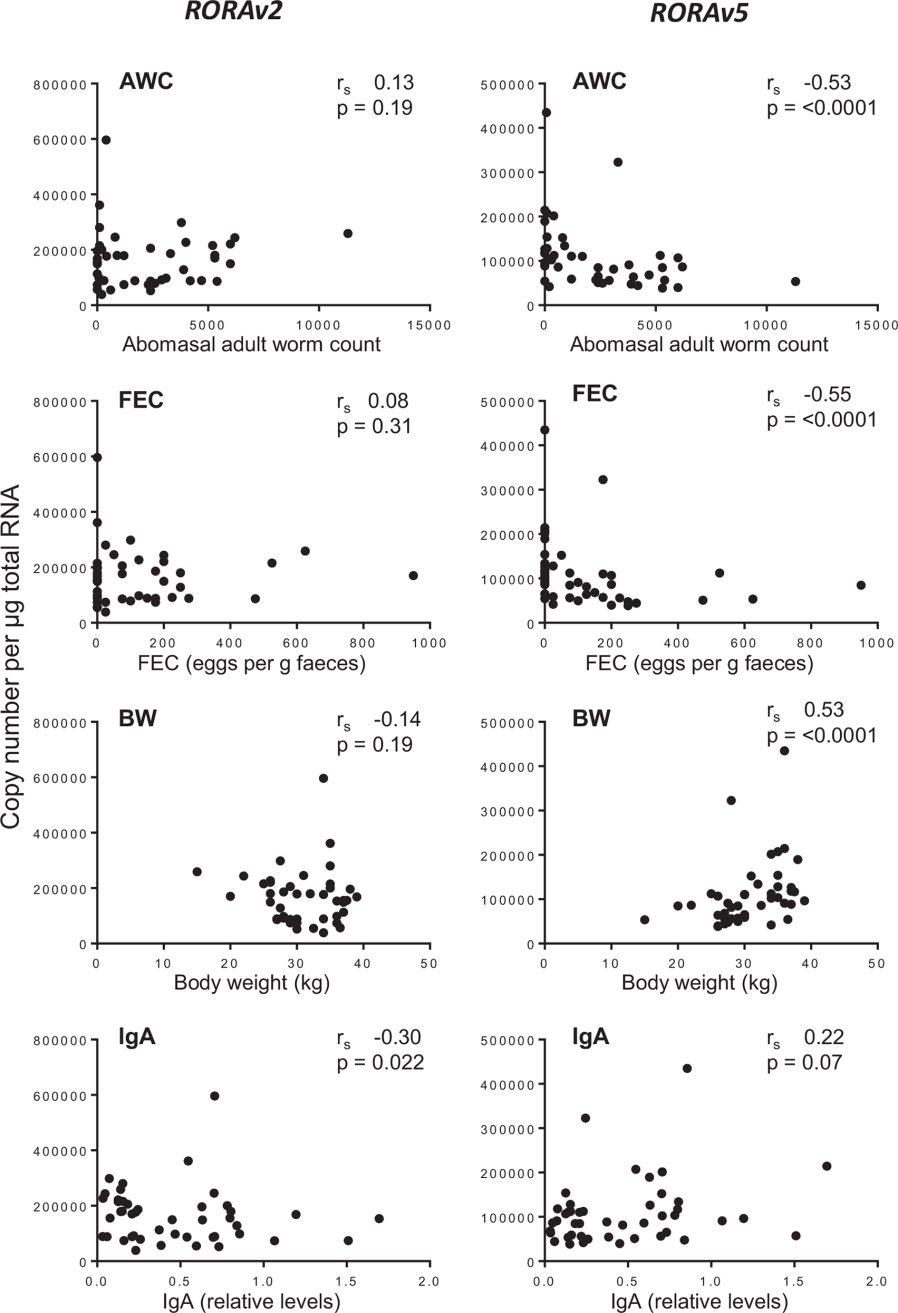
Correlation analysis of the phenotypic parameters and RORA transcript variants. Correlation of AWC, FEC, BW and IgA with *RORAv2 and RORAv5* copy number per μg total RNA in ALN of *T*. *circumcincta* infected sheep. r_s_—Spearman’s rank correlation coefficient.

Spearman’s rank analysis was used to quantify the correlation of expression levels of *RORAv2* and *RORAv5* in relation to the quantitative phenotypes, AWC, FEC, BW and IgA antibody levels (Fig 3). *RORAv2* expression levels were not significantly correlated with AWC, FEC or BW but were significantly negatively correlated with IgA (r_s_ -0.30, p = 0.022). In contrast *RORCv5* was significantly negatively correlated with AWC (r_s_ -0.53, p = <0.0001) and FEC (r_s_ -0.55, p = <0.0001), and significantly positively correlated with BW (r_s_ 0.53, p = <0.0001); but was not significantly correlated with IgA antibody (r_s_ 0.22, p = 0.07).

## Discussion

This study represents the first systematic attempt to assess the role of the four major immune system transcription factors in an ovine infectious disease context. Initial characterization of mRNA from abomasal lymph node failed to find any splice variants of sheep *TBX21*, identified two transcript variants each of *GATA3* and *RORC2*, and five transcript variants of *RORA*. *TBX21* has been unambiguously identified in relatively few species, including humans, cattle and mice, and in all these species only a single gene has been characterized.

The sequence difference between the two ovine *GATA3* variants is also found in the two variants of human *GATA3* with no reported phenotypic consequence. The deleted aa260 is not within either of the two conserved zinc-finger domains (aa264–286 and aa318–342) nor the functionally-critical DNA-binding YxKxHxxxRP motif (YYKLHNINRP) at aa345–354 [40] although it is close to the NH_2_-zinc finger and could possible affect protein structure. Only one *Bos taurus GATA3* sequence has been identified (NM_001076804.1) that encodes an identical protein (isoform X2) to full length sheep *GATA3*. There are also four predicted *Bos* transcript variants, *X1*, *X2* and *X3* encode isoform X2, and variant *X4* encodes a protein (isoform X1) identical to sheep *GATA3v1*.

Humans express two transcript variants of *RORC*; transcript variant 1 encodes full length *RORC* (isoform a or RORγ) of 518 amino acids with major functions in regulating embryo development as well as testicular and liver functions [41]. Transcript variant 2 (isoform b or RORγt) is 485 amino acids and has a major role in regulating Th17 T cells [27]. The two sheep *RORC2* variants are homologous to human transcript variant 2, and the 12 amino acid deletion in *RORC2v1* is within the predicted ligand binding domain [42]. One major *RORC2* variant also exists in humans; this has a deletion of exons 5–8, also within the ligand binding domain, and functions to suppress Th17 function by the repression of *IL17A* and *IL21* gene transcription [43].

Previous studies, using RT-qPCR of cytokine transcripts and whole genome microarrays, have shown that a strong Th2 response is associated with resistance and Th1/Th17 activation linked to susceptibility [16, 17]. However, comparison of resistant and susceptible animals showed that there was no differential expression of any of the three master regulators (or variants) that control T cell polarization, *TBX21*, *GATA3* or *RORC2*. A possible explanation for this is that the lymph nodes studied had been responding to *T*. *circumcincta* infection for 12 weeks and that the regulatory events controlling T cell polarization occurred much earlier in infection; during initial activation of T helper cells.

The identification of five *RORA* transcript variants in sheep is not an unusual finding, as several species express multiple *RORA* variants, including four in humans. All four human isoforms have identical zinc-finger DNA and ligand-binding domains but differ in their NH_2_-terminal sequences (A/B domains [44]) and it seems that these differences are associated with cellular expression levels [45] and determine functions [46, 47]. Indeed in mice, NH_2_-terminal truncated RORα isoforms are linked to cerebellar cell atrophy and the ‘staggerer’ phenotype [48]. The five sheep *RORA* variants also encode four distinct proteins. The derived protein sequences of *RORAv2* and *RORAv4* are identical; they possess the conserved ligand-binding domain but lack A/B domains and the DNA-binding domain. The *RORAv1* protein (RORαv1) contains both DNA- and ligand-binding domains and a 17 amino acid B domain immediately upstream of the DNA-binding domain, which is identical to that of human Rora isoform d (NP_599024.1). RORαv3 and RORαv5 possess both DNA- and ligand-binding domains and identical B domains, which is the same sequence as that of human isoform a, and distinct from that of RORαv1. RORαv3 possesses an upstream A domain of 13 amino acids and RORαv5 has an A domain of 44 amino acids, different to any human isoform.

Of the four measurable variants of *RORA* only *RORAv5* showed consistent differential expression in the resistant vs susceptible comparison and quantitative levels of *RORAv5* were highly significantly correlated with quantitative parameters of resistance. This is likely to be related to the length and identity of the A/B domains. Human Rora isoforms a and b have distinct A/B domains and display different binding specificities. The A/B domains of isoform a mediate almost complete nuclear localization [44] making it a strong transcription factor that activates a broad range of genes. In contrast the A/B domains of isoforms b, c and d mediate cytoplasmic localization and consequently they are weaker transcription factors with a narrower range of targets [46].

This does not fully explain the link between sheep *RORAv5* and parasite resistance. Analysis of the sheep immune response to *T*. *circumcincta* shows that resistance is correlated with the development of a Th2 response, including the production of IL-13 and up-regulated transcription of genes associated with wound healing [17]. In humans a major function of Rora isoform a (most similar to RORαv5) is the inhibition of NF-κB associated inflammation [37], and it is clear from previous studies in sheep that resistant animals are those with repressed inflammatory gene expression [16, 17]. RORα is also an essential transcription factor for nuocyte development in mice; these mediate Th2 polarization [29], optimize tissue repair [49] and consequently have a critical role in parasite expulsion. However, if the link between RORαv5 and parasite resistance is due to this ILC2 subset in sheep it does not involve the GATA-3^+^ ILC2 cells [49, 50] as this transcription factor is not differentially-expressed in parasite-resistant sheep. It is also known that RORα is an important accessory transcription factor for the development and function of Th17 cells [28], but the master regulator of these cells is RORγt which, like GATA-3, is not differentially-expressed; furthermore Th17 activation seems to be more associated with susceptibility than resistance [16].

Analysis of *RORAv2* expression showed no correlation with AWC, FEC or BW and a low but significant negative correlation with IgA, i.e. a positive correlation with susceptibility. RORαv2 possesses neither the A/B nor DNA-binding domains, but does have the ligand-binding domain. Both *RORAv2* and *RORAv5* are expressed at high levels only after infection. however *RORAv5* showed a 13 fold up-regulation in resistant animals, but only a 7 and 6 fold increase in the intermediate and susceptible groups. In contrast, the infection-associated increase in *RORAv2* expression was similar in all three groups. It is possible that RORαv2, which is found at higher levels than RORαv5 in all infected lambs, could compete with RORαv5 for ligand binding and inhibit its function in those animals with lower levels of *RORAv5*, with consequent pathological effects in susceptible lambs.

In conclusion, we have identified transcript variants of the master regulators of T cell immunity in sheep. There are two variants of *GATA3* and *RORC2*, five variants of *RORA* and a single *TBX21* transcript. Analysis of expression of each variant in sheep with defined resistance status to the gastrointestinal parasite *T*. *circumcincta* showed that only *RORAv5* is differentially expressed in resistant compared to susceptible sheep, with expression levels that correlate with the phenotypic parameters of resistance. The largest variant of *RORA* (*RORAv5*) seems to be important in controlling the protective immune response to *T*. *circumcincta* infection in sheep.

## Supporting Information

S1 Fig Agarose gel electrophoresis of sheep *RORC1* and *RORC2*. RT-PCR products using: (A) *RORC1* primers, Lanes 1 and 2 mRNA from liver; Lane 3 mRNA from liver (no RT control); Lane 4 mRNA from lymph node; (B) *RORC2* primers, Lane 1 mRNA from lymph node (no RT control); Lanes 2 and 3 mRNA from lymph node.
(PDF)

S2 Fig *Ovis aries GATA3* transcript variants. (A) 5’ nucleotide sequences (LN848231 and LN848232). (B) derived NH_2_-protein sequences (CRI68167.1 and CRI68168.1).
(PDF)

S3 Fig *Ovis aries RORC2* transcript variants. (A) 5’ nucleotide (LN848233 and LN848234). (B) derived NH_2_-protein sequences (CRI68169.1 and CRI68170.1).
(PDF)

S4 Fig *Ovis aries RORA* nucleotide sequences. 5’ nucleotide sequences of RORA transcript variants (LN848235, LN848236, LN848237, LN848238, LN848239).
(PDF)

S5 Fig *Ovis aries RORA* transcript variants. *RORAv1*, *v2*, *v3*, *v4* and *v5* intron and exon structures, mapped to Oarv3.1 genome assembly.
(PDF)

S1 Table Quantitative phenotypic data and normalized copy numbers of *RORAv2* and *RORAv5*, in ALN of Scottish Blackface lambs persistently infected with *T*. *circumcincta*. (PDF)

S2 Table (A) Primer sets for amplification of sheep transcription factors. (B) Primer sequences for 5’ and 3’ RACE. (C) Primer sets for RT-qPCR with optimized parameters and volumes.
(PDF)
