# Correction: Variations in T cell transcription factor sequence and expression associated with resistance to the sheep nematode *Teladorsagia circumcincta*

**DOI:** 10.1371/journal.pone.0152396

**Published:** 2016-03-29

**Authors:** Hazel Wilkie, Anton Gossner, Stephen Bishop, John Hopkins

There are three errors in the Abstract section of the manuscript, specifically lines 29–34. The correct text should be: Absolute RT-qPCR on 29 all 45 animals identified RORAv5 as being significantly differentially-expressed (p = 0.038) 30 between resistant, intermediate and susceptible groups; RORAv2 was not differentially- 31 expressed (p = 0.77). Spearman’s rank analysis showed that RORAv5 transcript copy number 32 was significantly negatively correlated with parameters of susceptibility, AWC and FEC; and 33 was positively correlated with BW. RORAv2 was not correlated with AWC, FEC or BW but 34 was significantly negatively correlated with IgA antibody levels.
