# Supplementary figures and images for: Variations in T Cell Transcription Factor Sequence and Expression Associated with Resistance to the Sheep Nematode Teladorsagia circumcincta
Source: PLoS One. 2016 Feb 18;11(2):e0149644. doi: 10.1371/journal.pone.0149644 (PMC4759366; doi:10.1371/journal.pone.0149644)

A. RORC1

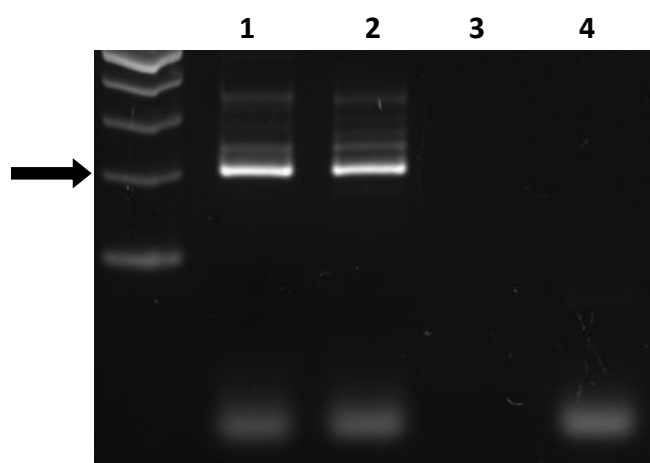

B. RORC2

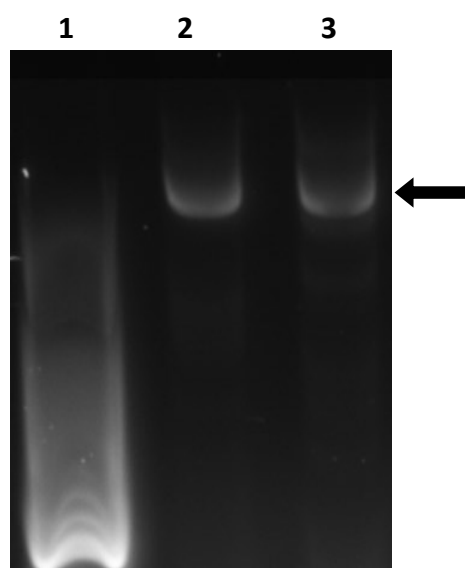

Supplement: S1 Fig — RT-PCR products using: (A) RORC1 primers, Lanes 1 and 2 mRNA from liver; Lane 3 mRNA from liver (no RT control); Lane 4 mRNA from lymph node; (B) RORC2 primers, Lane 1 mRNA from lymph node (no RT control); Lanes 2 and 3 mRNA from lymph node. (PDF) [file pone.0149644.s001.pdf]

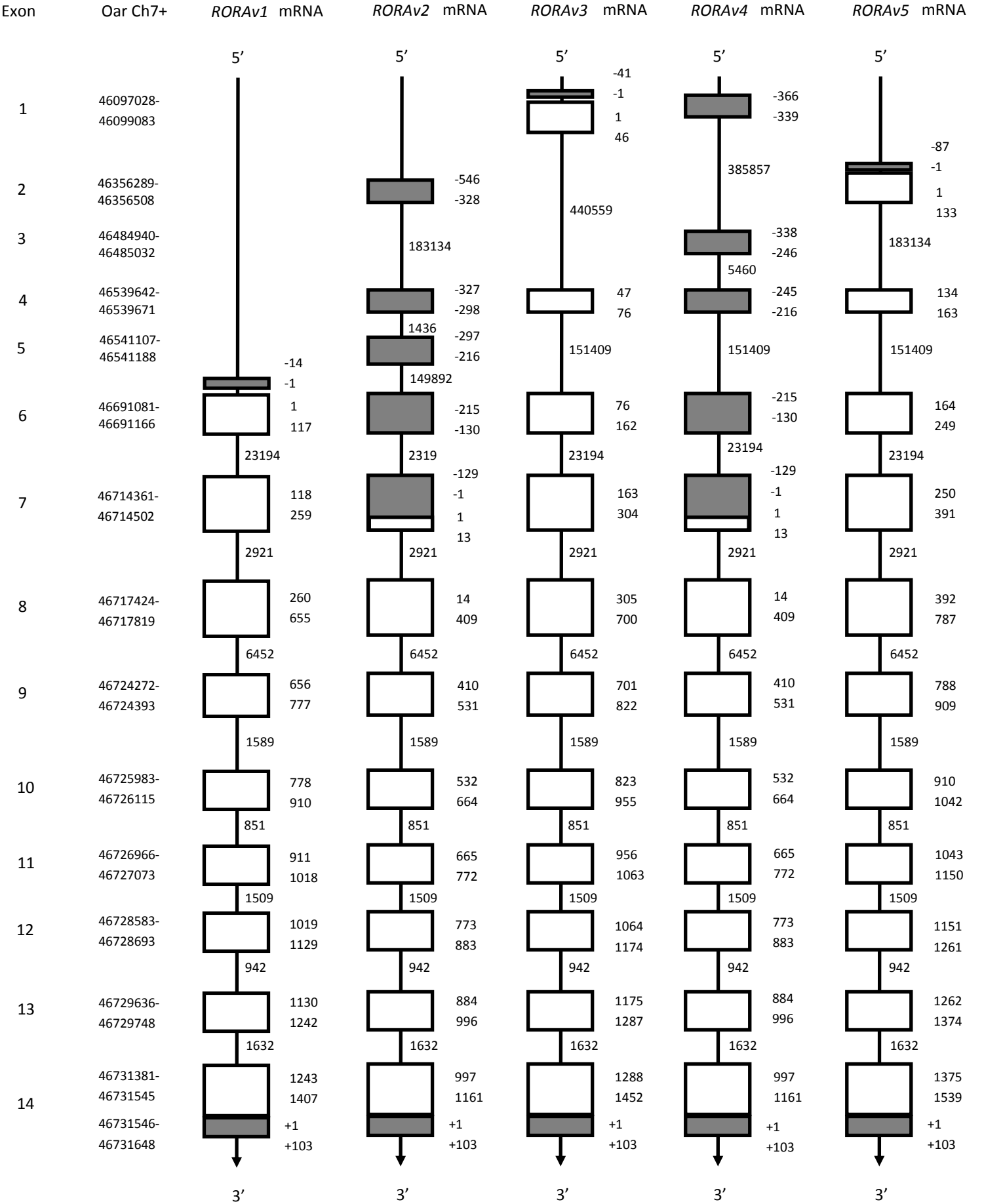

Translation start sites

RORAv1: unmapped in Oar v3.1.

RORAv2: 46714490. RORAv3: 4609038. RORv4: 46714490. RORAv5: 4635376

Supplement: S5 Fig — RORAv1, v2, v3, v4 and v5 intron and exon structures, mapped to Oarv3.1 genome assembly. (PDF) [file pone.0149644.s005.pdf]
