# Supplementary material for: Variations in T Cell Transcription Factor Sequence and Expression Associated with Resistance to the Sheep Nematode Teladorsagia circumcincta
Source: PLoS One. 2016 Feb 18;11(2):e0149644. doi: 10.1371/journal.pone.0149644 (PMC4759366; doi:10.1371/journal.pone.0149644)
Supplement: S2 Fig — (A) 5’ nucleotide sequences (LN848231 and LN848232). (B) derived NH2-protein sequences (CRI68167.1 and CRI68168.1). (PDF) [file pone.0149644.s002.pdf]

(A) *Ovis aries GATA3* 5’ nucleotide sequences (LN848231 and LN848232)

```
GATA3        GAGGCCCAAGGCGAGATCCAGCACAGAAGGCCGGGAGTGTGTGAACTGCGGGGCGACATC   840
GATA3v1      GAGGCCCAAGGCGAGATCCAGCACA---GGCCGGGAGTGTGTGAACTGCGGGGCGACATC   837
             *************************   ********************************
```

(B) *Ovis aries* GATA-3 derived NH_2-protein sequences (CRI68167.1 and CRI68168.1)

```
Gata3        GGSPTGFGCKSRPKARSSTEGRECVNCGATSTPLWRRDGTGHYLCNACGLYHKMNGQNRP   300
Gata3v1      GGSPTGFGCKSRPKARSST-GRECVNCGATSTPLWRRDGTGHYLCNACGLYHKMNGQNRP   299
             ******************  ****************************************
```
