# Supplementary material for: Variations in T Cell Transcription Factor Sequence and Expression Associated with Resistance to the Sheep Nematode Teladorsagia circumcincta
Source: PLoS One. 2016 Feb 18;11(2):e0149644. doi: 10.1371/journal.pone.0149644 (PMC4759366; doi:10.1371/journal.pone.0149644)
Supplement: S3 Fig — (A) 5’ nucleotide (LN848233 and LN848234). (B) derived NH2-protein sequences (CRI68169.1 and CRI68170.1). (PDF) [file pone.0149644.s003.pdf]

(A) Ovis aries *RORC2* nucleotide sequences (LN848233 and LN848234)

```
RORC2          CCTACAACGCTGACAACAACACAGTCTTTTTTGAAGGCAAATACGGTGGCGTGGAGCTGT  1260
RORC2v1        CCTACAACGCTGACAACAACACAGTCTTTTTTGAAG------------------------  1236
               ************************************

RORC2          TCCGAGCCTTGGGCTGCAGTGAACTCATCAGCTCCATCTTTGACTTCTCCCGCTCCCTGA  1320
ROR2Cv1        ------------GCTGCAGTGAACTCATCAGCTCCATCTTTGACTTCTCCCGCTCCCTGA  1284
                           ************************************************
```

(B) *Ovis aries* RORγt 2 derived protein sequences (CRI68169.1 and CRI68170.1)

```
Rorγ2          HRLTEAIQYVVEFAKRLPGFMELCQNDQIVLLKAGAMEVVLVRMCRAYNADNNTVFFEGK  360
Rorγ2v1        HRLTEAIQYVVEFAKRLPGFMELCQNDQIVLLKAGAMEVVLVRMCRAYNADNNTVFFE--  358
               **********************************************************

Rorγ2          YGGVELFRALGCSELISSIFDFSRSLSALRFSEDEIALYTALVLINANRPGLQEKRKVEQ  420
Ror2γv1        ----------GCSELISSIFDFSRSLSALRFSEDEIALYTALVLINANRPGLQEKRKVEQ  408
                         **************************************************
```
