# Supplementary material for: Variations in T Cell Transcription Factor Sequence and Expression Associated with Resistance to the Sheep Nematode Teladorsagia circumcincta
Source: PLoS One. 2016 Feb 18;11(2):e0149644. doi: 10.1371/journal.pone.0149644 (PMC4759366; doi:10.1371/journal.pone.0149644)
Supplement: S4 Fig — 5’ nucleotide sequences of RORA transcript variants (LN848235, LN848236, LN848237, LN848238, LN848239). (PDF) [file pone.0149644.s004.pdf]

Ovis aries RORA 5’ nucleotide sequences (LN848235, LN848236, LN848237, LN848238, LN848239)

```
RORAv1      ------------------------------------------------------------   0
RORAv2      CGATTCCTCGGGGTTTCACAACCTAAGAGGTCTGGGAGGGCACCTTGCAGTGCCTAAGTC   60
RORAv3      ------------------------------------------------------------   0
RORAv4      ------------------------------------------------------------   0
RORAv5      CGATTCCTCGGGGTTTCACAACCTAAGAGGTCTGGGAGGGCACCTTGCAGTGCCTAAGTC   60


RORAv1      ------------------------------------------------------------   0
RORAv2      TCCACGAGTGTTCAGAGCGGACATAAAATGTACAGAGTCTTTAGACAAGCGG---GCTTT   117
RORAv3      -------------TGCGCAGA---------------------------------------   8
RORAv4      --------------ACACTGACATGGACTGAA-GGAG----TAGAAAAGAAGGCAGCTTT   41
RORAv5      TCCACGAGTGTTCAGAGCGGACATAAAATGTACGGAGTCTTTAGACAAGCGG---GCTTT   117


RORAv1      ------------------------------------------------------------   0
RORAv2      CTGTGGATGGGATCCGCCTCTGGGAGGCCAGGG-AAAAGACAAAAACAAGTGTTTCCTGC   176
RORAv3      CAGAGCT---ATTCCAGCACCAG-----CAGAGGG---------------TTATCCATGC   45
RORAv4      CTTCTGGTGTCGTCAGCCTCTTGCAT--CTGGGTCC--------------AGGTCCCTTC   85
RORAv5      CTGTGGATGGGATCCGCCTCTGGGAGGCCAGGGGAAAAGACAAAAACAAGTGTTTCCTGC   177


RORAv1      ------------------------------------------------------------   0
RORAv2      AGTTCTCTGCTGCAGTTGCTAACAGAGAGT---CACTCGACGCTTGGTATCTCAGTAACA   233
RORAv3      CAGTGTCCAGTGAGG----GAACTGAGAGAAGGCGGCATGATCCAGGTATCTCAGTAACA   101
RORAv4      AAGGTTAATCA---G----AATCAGAA---GTTCAGAGAGCTGCAGGTATCTCAGTAACA   135
RORAv5      AGTTCTCTGCTGCAGTTGCTAACAGAGAGT---CACTCGACGCTTGGTATCTCAGTAACA   234


RORAv1      -------------------------------------------TCACCGCG---------   8
RORAv2      AAGAAGACCCATACATGGCAGGCATGGTACAGAGAGCTTCCTTGCACAGCCTTGCCCATG   293
RORAv3      AAGAAGACCCATACAT--------------------------------------------   117
RORAv4      AAGAAGACCCATACAT--------------------------------------------   151
RORAv5      AAGAAGACCCATACAT--------------------------------------------   250


RORAv1      -GCTTAAATGATGTATTTTGTGATCGCAGCGATGAAAGCTCAAATTGAAATTATTCCATG   67
RORAv2      AGCTCGTCCCTGGCCTGGTGGGACCCACTCTAGAGGTGCTCAAATTGAAATTATTCCATG   353
RORAv3      --------------------------------------CTCAAATTGAAATTATTCCATG   139
RORAv4      --------------------------------------CTCAAATTGAAATTATTCCATG   173
RORAv5      --------------------------------------CTCAAATTGAAATTATTCCATG   272
                                                  **********************

RORAv1      CAAGATCTGTGGAGACAAATCATCAGGAATCCATTACGGTGTCATTACATGTGAAGGCTG   127
RORAv2      CAAGATCTGTGGAGACAAATCATCAGGAATCCATTACGGTGTCATTACATGTGAAGGCTG   413
RORAv3      CAAGATCTGTGGAGACAAATCATCAGGAATCCATTACGGTGTCATTACATGTGAAGGCTG   199
RORAv4      CAAGATCTGTGGAGACAAATCATCAGGAATCCATTACGGTGTCATTACATGTGAAGGCTG   233
RORAv5      CAAGATCTGTGGAGACAAATCATCAGGAATCCATTACGGTGTCATTACATGTGAAGGCTG   332
            ************************************************************

RORAv1      CAAGGGCTTTTTCAGGAGAAGTCAGCAAAGCAATGCCACCTACTCCTGTCCTCGTCAAAA   187
RORAv2      CAAGGGCTTTTTCAGGAGAAGTCAGCAAAGCA8     473
RORAv3      CAAGGGCTTTTTCAGGAGAAGTCAGCAAAGCAATGCCACCTACTCCTGTCCTCGTCAAAA   259
RORAv4      CAAGGGCTTTTTCAGGAGAAGTCAGCAAAGCAATGCCACCTACTCCTGTCCTCGTCAAAA   293
RORAv5      CAAGGGCTTTTTCAGGAGAAGTCAGCAAAGCAATGCCACCTACTCCTGTCCTCGTCAAAA   392
            ************************************************************

RORAv1      GAACTGTTTGATTGATCGGACCAGTAGAAACCGCTGCCAGCACTGTCGATTACAGAAATG   247
RORAv2      GAACTGTTTGATTGATCGGACCAGTAGAAACCGCTGCCAGCACTGTCGATTACAGAAATG   533
RORAv3      GAACTGTTTGATTGATCGGGCCAGTAGAAACCGCTGCCAGCACTGTCGATTACAGAAATG   319
RORAv4      GAACTGTTTGATTGATCGGACCAGTAGAAACCGCTGCCAGCACTGTCGATTACAGAAATG   353
RORAv5      GAACTGTTTGATTGATCGGACCAGTAGAAACCGCTGCCAGCACTGTCGATTACAGAAATG   452
            *******************.****************************************
```

RORAv1	**CCTTGCCGTGGGGATGTCTCGAGATGCTGTGAAATTTGGCCGCATGTCGAAAAAGCAGAG**	307
RORAv2	CCTTGCCGTGGGG**ATGTCTCGAGATGCTGTGAAATTTGGCCGCATGTCGAAAAAGCAGAG**	593
RORAv3	**CCTTGCCGTGGGGATGTCTCGAGATGCTGTGAAATTTGGCCGCATGTCGAAAAAGCAGAG**	379
RORAv4	CCTTGCCGTGGGG**ATGTCTCGAGATGCTGTGAAATTTGGCCGCATGTCGAAAAAGCAGAG**	413
RORAv5	**CCTTGCCGTGGGGATGTCTCGAGATGCTGTGAAATTTGGCCGCATGTCGAAAAAGCAGAG**	512
	**	

Bold; coding region
