# Supplementary material for: Variations in T Cell Transcription Factor Sequence and Expression Associated with Resistance to the Sheep Nematode Teladorsagia circumcincta
Source: PLoS One. 2016 Feb 18;11(2):e0149644. doi: 10.1371/journal.pone.0149644 (PMC4759366; doi:10.1371/journal.pone.0149644)
Supplement: S1 Table — (PDF) [file pone.0149644.s006.pdf]

Table S1. Quantitative phenotypic data and normalized copy numbers of *RORAv2* and *RORAv5* , in ALN of Scottish Blackface lambs persistently infected with *T. circumcincta*

Sheep	Infection rank[1]	AWC[2]	FEC[3]	BW (kg)	IgA[4]	RORA ex2[5]	RORA ex5[5]
Resistant group rank 1 - 15; mean AWC = 59, mean FEC = 1.7.							
LN_92	1	0	0	39	1.195	168519	96657
LN_100	2	0	0	38	0.63	196483	189477
LN_21	3	0	0	37.5	0.798	155351	116701
LN_20	4	0	0	37	0.633	149323	126319
LN_58	5	0	0	37	0.077	155566	118300
LN_50	6	0	0	37	0.373	113207	88681
LN_110	7	0	0	36.5	0.384	56920	54460
LN_54	8	0	0	36	1.695	153364	214298
LN_116	9	0	0	36	1.066	73569	91144
LN_25c	10	80	0	36	0.856	98063	434974
LN_155	11	100	0	35	0.126	214712	154054
LN_52	12	100	0	35	0.547	361605	207413
LN_34	13	100	25	35	0.154	280432	128104
LN_184	14	200	0	35	0.782	200339	103692
LN_123	15	300	0	34	0.706	88709	102318
Intermediate group rank 16 - 30; mean AWC = 1508, mean FEC = 87.							
LN_10	16	400	0	34	0.706	596542	201603
LN 193	17	420	75	34	0.232	177096	112438
LN_102	18	200	25	34	0.232	39114	41800
LN_40	19	600	0	32.5	0.596	55246	86086
LN_12	20	900	0	32	0.804	179662	134129
LN_125	21	800	50	31	0.703	245492	152256
LN_62	22	1200	0	30	0.142	179077	110814
LN_172	23	1700	175	30	0.21	88088	110107
LN_181	24	1200	25	30	0.161	74519	58825
LN_165	25	2400	0	30	0.732	52622	65160
LN_19	26	2300	175	29	1.51	74498	57209
LN_8	27	2400	475	29	0.539	87253	51240
LN_138	28	2400	75	29	0.183	205677	84670
LN_48	29	2600	100	29	0.259	79135	49792
LN_30	30	3100	125	28	0.468	97691	81293
Susceptible group rank 31 - 45; mean AWC = 5167, mean FEC = 288.							

LN_82	31	3300	175	28	0.245	186062	322638
LN_190	32	2900	225	28	0.219	91856	55890
LN_178	33	3800	100	27.5	0.073	298578	91203
LN_59	34	3900	250	27.5	0.84	128348	47804
LN_191	35	4200	275	27	0.06	88503	44378
LN_65	36	4700	150	27	0.033	88682	68037
LN_60	37	5400	75	27	0.697	86479	56608
LN_119	38	5300	250	26	0.151	180266	38623
LN_131	39	4000	125	26	0.035	227123	63791
LN_28	40	6000	200	26	0.126	220789	106895
LN_114	41	6000	200	26	0.451	149347	39679
LN_38	42	5200	525	25	0.155	215840	112128
LN_173	43	6200	200	22	0.047	243581	86390
LN_183	44	5300	950	20	0.209	170065	84659
LN_109	45	11300	625	15	0.141	258809	53542
Uninfected control group, mean AWC = 0, mean FEC = 0							
LN11	Control	0	0	35.5	<0.02	15654	9470
LN39	Control	0	0	36	<0.02	12865	13074
LN47	Control	0	0	36	<0.02	23688	9774
LN57	Control	0	0	34.5	<0.02	0	11064
LN81	Control	0	0	29	<0.02	5767	7300
LN124	Control	0	0	27.5	<0.02	44546	10192
LN130	Control	0	0	30	<0.02	12532	4312
LN146	Control	0	0	36	<0.02	16611	11114
LN182	Control	0	0	33	<0.02	0	8062
LN192	Control	0	0	32	<0.02	11697	28717

[1] Ranked on the basis of both AWC and FEC

[2] Adult worm count, total numbers of adult worms in the abomasal contents at post mortem.

[3] Fecal egg counts per g feces at post mortem

[4] Relative levels of serum IgA

[5] Copy numbers per µg in total RNA
