# Supplementary material for: Variations in T Cell Transcription Factor Sequence and Expression Associated with Resistance to the Sheep Nematode Teladorsagia circumcincta
Source: PLoS One. 2016 Feb 18;11(2):e0149644. doi: 10.1371/journal.pone.0149644 (PMC4759366; doi:10.1371/journal.pone.0149644)
Supplement: S2 Table — (A) Primer sets for amplification of sheep transcription factors. (B) Primer sequences for 5’ and 3’ RACE. (C) Primer sets for RT-qPCR with optimized parameters and volumes. (PDF) [file pone.0149644.s007.pdf]

Table S2

(A) Primer sets for amplification of sheep transcription factors

Gene	Primer sequence (5'to 3')	Product size (bp)	Anneal temp (°C)
RORC2	F: CCCGGCCCAGAAGCATTG<br>R: TCTCAGCGTTTGTTCACCCA	1790	55
GATA3	F: GCGAGAGGGCGCGAG<br>R: ACACAGATCCGTCGGGTTTC	1477	55 - 58
RORAv1	F: CTCCTCACCGCGGCTTAAAT<br>R: TGCGCAGACAGAGCTATTCC	1546	52
RORAv2 5'	F: CGATTCCTCGGGGTTTCACA<br>R: TTTGAGCACCTCTAGAGTGGG	338	60
RORAv2 3'	F: ACATGGCAGGCATGGTACAGAG<br>R: GCGGCCAAATTTCACAGC	331	60
RORAv3	F: TGCGCAGACAGAGCTATTCC<br>R: GCGGCCAAATTTCACAGC	390	55
RORAv4	F: ACACTGACATGGACTGAAGG<br>R: GCGGCCAAATTTCACAGC	427	52
RORAv5	F: CGATTCCTCGGGGTTTCACA<br>R: GCGGCCAAATTTCACAGC	275	60
RORC1	F: CCACAGAGACACCACCGAGC<br>R: CTGACGGGTGCAGGAGTAGG	198	60

(B) Primer sequences for 5' and 3' RACE

Gene	Primer sequence	Product size (bp)	Anneal temp (°C)
GATA3 5'	CCGGTTCTGTCCGTTCATCT	900	55
GATA3 3'	CACCCGGACACTCACCAC	1260	55
RORA 5'	GCCACGTTATCTGCTGGAGCT	980	58
RORA 5' nested end	GCCTGATGCTGGTGTGTAGT	837	58
RORC 5' end	CTTCCCTGACGGAGATTGAGC	840	60

(C) Primer sets for RT-qPCR with optimized parameters and volumes

Gene	Primer sequences	RT-qPCR parameters (all 40 cycles)	Primer (10μM)
TBX21	F: CCTGTTGTGGTCCAAGTTC<br>R: CGGTAATGGCTGGTGGGCTC	95° 10 min, (95° 10 s, 60° 45 s)	0.5 μl
GATA3	F: CCACAAGATGAACGGACAG<br>R: GGCATTTCTTCTCCACAGAGTCGT	95° 10 min, (95° 15 s, 62° 45 s)	1.0
GATA3v1	F: GCGAGATCCAGCACAGGCC<br>R: GTTCTGTCCGTTCATCTTGTGG	95° 10 min, (95° 15 s, 60° 15 s, 72° 30 s)	0.5
RORC2	F: CGCTGTGCCCACCGACTCACCGAG<br>R: TGACCAGCACCACTTCCATG	95° 10 min, (95° 15 s, 62° 45 s)	1.0
RORC2v1	F: TTGAAGGCTGCAGTGAACTC<br>R: GGCATTGATGAGCACGAGG	95° 10 min, (95° 15 s, 63° 45 s)	0.25
RORAv1	F: GTGATCGCAGCGATGAAAGC<br>R: CCTTGCAGCCTTCACATGTAATG	95° 10 min, (95° 10 s, 60° 45 s)	0.5
RORAv2	F: AGTTCTCTGCTGCAGTTGCTAAC R: GACGAGCTCATGGGCAAGG	95° 10 min, (95° 15 s, 62° 45 s)	0.35
RORAv3	F: TGCGCAGACAGAGCTATTCC<br>R: TGGGTCTTCTTTGTTACTGAGATACC	95° 10 min, (95° 15 s, 62° 15 s, 72° 30 s)	0.5
RORAv4	F: GCAGCTTTCTTCTGGTGTCGTC<br>R: TGGGTCTTCTTTGTTACTGAGATACC	95° 10 min, (95° 10 s, 60° 45 s)	0.5
RORAv5	F: GGCTTTCTGTGGATGGGATC<br>R: TGGGTCTTCTTTGTTACTGAGATACC	95° 10 min, (95° 10 s, 60° 45 s)	0.3
